# Supplementary material for: End Sequence Analysis Toolkit (ESAT) expands the extractable information from single-cell RNA-seq data
Source: Genome Res. 2016 Oct;26(10):1397–410. doi: 10.1101/gr.207902.116 (PMC5052061; doi:10.1101/gr.207902.116)
Supplement: Supplemental Material [file supp_gr.207902.116_Supp_Materials.tar.gz › ESAT-esat_v0.1/doc/html/interfacebroad_1_1pda_1_1seq_1_1segmentation_1_1_alignment_data_model.html]

ESAT: broad.pda.seq.segmentation.AlignmentDataModel Interface Reference


ESAT


- Main Page
- Related Pages
- Packages
- Classes
- Files

- Class List
- Class Index
- Class Hierarchy
- Class Members

All Classes Namespaces Files Functions Variables Enumerator Pages

- broad
- pda
- seq
- segmentation
- AlignmentDataModel

Public Member Functions |
List of all members

broad.pda.seq.segmentation.AlignmentDataModel Interface Reference

Inheritance diagram for broad.pda.seq.segmentation.AlignmentDataModel:

[legend]

|  |  |
| --- | --- |
| Public Member Functions | |
| CloseableIterator< Alignment > | getAnnotationOverlappingRegion (Annotation align) throws IOException |
|  | |
| Map< String, Integer > | getChromosomeLengths () |
|  | |
| int | getChromosomeLength (String chr) |
|  | |
| double | getCounts (String chr) throws IOException |
|  | |
| double | getCountsPerAlignment (Annotation first, int EF) throws IOException |
|  | |
| double | getCountsPerAlignment (Annotation align, IntervalTree< Alignment > tree, int EF) |
|  | |
| double | getCountsPerAlignment (Gene gene, IntervalTree< Alignment > tree, int EF) |
|  | |
| double | getCountsPerAnnotationtranded (Gene gene, IntervalTree< Alignment > tree, int EF, String orientation) |
|  | |
| double | getCountsPerAlignment (Annotation[] Annotation, IntervalTree< Alignment > tree, int EF) |
|  | |
| double | getCountsPerAlignmentFullyContained (Gene gene, IntervalTree< Alignment > tree, int EF) |
|  | |
| double | getCountsPerAlignment (Annotation[] Annotation, int EF) throws IOException |
|  | |
| double | getCountsPerAlignment (Annotation align, Map< String, IntervalTree< Annotation >> goodExonTree, IntervalTree< Alignment > tree, int extensionFactor) |
|  | |
| int | getCountsWithinExons (Annotation align, Iterator< Alignment > iter, int EF) |
|  | |
| int | getCountsWithinExons (Annotation align, IntervalTree< Alignment > tree, int EF) |
|  | |
| double | getCountsOfUniqueOverlappers (Annotation target, Annotation exclude, IntervalTree< Alignment > tree, int EF) |
|  | |
| double | getCountsOfUniqueOverlappers (Annotation target, Gene exclude, IntervalTree< Alignment > tree, int EF) |
|  | |
| double | getCountsOnBase (String chr, int index) throws IOException |
|  | |
| int | getBasesCovered (Annotation record, int EF) throws IOException |
|  | |
| double | getNumberOfSplicedReads (Annotation align) throws IOException |
|  | |
| GeneCounts | getGeneCounts (Gene gene, int extensionFactor) throws IOException |
|  | |
| double | getBasesCoveredPerAlignment (Gene gene, IntervalTree< Alignment > tree, int EF) |
|  | |
| Iterator< Annotation > | getExonAnnotationOverlappingRegion (Annotation align) throws IOException |
|  | |
| Gene | getPeak (Gene gene, Gene startCodon, IntervalTree< Alignment > tree, int EF) |
|  | |
| Collection< Annotation > | getOverlappingRegions (String chr) throws IOException |
|  | |
| IntervalTree< Alignment > | getIntervalTree (String chr, int start, int end) throws IOException |
|  | |
| IntervalTree< Alignment > | getIntervalTreeCached (String chr, int start, int end) throws IOException |
|  | |
| Gene | updateGeneByFirstCounts (Gene gene, IntervalTree< Alignment > tree, int EF) |
|  | |
| Map< Annotation, Integer > | getSplicedReads (Annotation align, int minIntronSize, int maxIntronSize) throws IOException |
|  | |
| Map< Annotation, Integer > | getSplicedReads (Annotation region, Collection< Predicate< Annotation >> filters) throws IOException |
|  | |
| Map< Annotation, Integer > | getSplicedReads (Annotation region) throws IOException |
|  | |
| Map< Annotation, Integer > | getSplicedReads (Annotation align, final int minIntronSize, final int maxIntronSize, int minNumIntrons, Sequence chrSeq) throws IOException |
|  | |
| Map< Annotation, Integer > | getSplicedReads (Annotation region, Collection< Predicate< Annotation >> filters, int minNumIntrons) throws IOException |
|  | |
| Map< Annotation, Integer > | getSplicedReads (Annotation region, Collection< Predicate< Annotation >> filters, int minNumIntrons, Sequence chrSeq) throws IOException |
|  | |
| Map< Annotation, Integer > | getSplicedReads (Annotation region, Predicate< Annotation > filter) throws IOException |
|  | |
| Map< Annotation, Integer > | getSplicedReads (int minIntronSize, int maxIntronSize) throws IOException |
|  | |
| double | getSpliceWeightFactor () |
|  | |
| Collection<?extends Annotation > | getSplicedReadExons (String chr) throws IOException |
|  | |
| CloseableIterator< Alignment > | getReadIterator () |
|  | |
| CloseableIterator< Alignment > | getReadIterator (Annotation region) throws IOException |
|  | |
| CloseableIterator< Alignment > | getChromosomeReadIterator (String chromosome) throws IOException |
|  | |
| double | getTotalNumberOfStrandedReads () |
|  | |
| double | getScorePerAlignmentFromCache (Gene window, IntervalTree< Alignment > chunkAlignmentTree, int extensionFactor) |
|  | |
| void | resetTreeCache () |
|  | |
| int | getChunkStart () |
|  | |
| int | getChunkEnd () |
|  | |
| IntervalTree< Alignment > | getIntervalTreeTruncatedCached (String chromosome, int start, int end) throws IOException |
|  | |
| IntervalTree< Annotation > | getFullIntervalTreeAsAnnotation (String chr) throws IOException |
|  | |
| double | getMinimumMappingQuality () |
|  | |
| void | setChunkSize (int chunkSize) |
|  | |
| void | setNegativeStranded () |
|  | |
| void | setPositiveStranded () |
|  | |
| void | setSecondRead () |
|  | |
| void | setFirstRead () |
|  | |
| void | setNormalizationStrategy (ReadCountNormalizer normalizer) |
|  | |
| void | setMinimumMappingQuality (double minimumMapQuality) |
|  | |
| boolean | passes (Gene gene, IntervalTree< Alignment > tree, int EF, double cutoff) |
|  | |
| long | passesCutoff (Annotation align, IntervalTree< Alignment > tree, int EF, double threshold) |
|  | |
| boolean | hasNextExon (String chr) throws IOException |
|  | |
| void | restartIterator () |
|  | |
| void | resetTreeCache (String chr) |
|  | |
| void | clearFullIntervalTreeAsAlignmentsCached (String chr) |
|  | |
| boolean | isStranded () |
|  | |
| void | unsetStranded () |
|  | |
| boolean | isPositiveStranded () |
|  | |
| boolean | isNegativeStranded () |
|  | |
| String | getModelFilePath () |
|  | |
| void | setExtensionFactor (int factor) |
|  | |

## Member Function Documentation

|  |  |  |  |  |  |
| --- | --- | --- | --- | --- | --- |
| void broad.pda.seq.segmentation.AlignmentDataModel.clearFullIntervalTreeAsAlignmentsCached | ( | String | *chr* | ) |  |

Implemented in broad.pda.seq.segmentation.GenericAlignmentDataModel.

|  |  |  |  |  |  |
| --- | --- | --- | --- | --- | --- |
| CloseableIterator<Alignment> broad.pda.seq.segmentation.AlignmentDataModel.getAnnotationOverlappingRegion | ( | Annotation | *align* | ) | throws IOException |

Implemented in broad.pda.seq.segmentation.GenericAlignmentDataModel.

|  |  |  |  |
| --- | --- | --- | --- |
| int broad.pda.seq.segmentation.AlignmentDataModel.getBasesCovered | ( | Annotation | *record*, |
|  |  | int | *EF* |
|  | ) |  | throws IOException |

Implemented in broad.pda.seq.segmentation.GenericAlignmentDataModel.

|  |  |  |  |
| --- | --- | --- | --- |
| double broad.pda.seq.segmentation.AlignmentDataModel.getBasesCoveredPerAlignment | ( | Gene | *gene*, |
|  |  | IntervalTree< Alignment > | *tree*, |
|  |  | int | *EF* |
|  | ) |  |  |

Implemented in broad.pda.seq.segmentation.GenericAlignmentDataModel.

|  |  |  |  |  |  |
| --- | --- | --- | --- | --- | --- |
| int broad.pda.seq.segmentation.AlignmentDataModel.getChromosomeLength | ( | String | *chr* | ) |  |

Get length of a chromosome

Parameters
:   |  |  |
    | --- | --- |
    | chr |  |

Returns

Implemented in broad.pda.seq.segmentation.GenericAlignmentDataModel.

Here is the caller graph for this function:

|  |  |  |  |  |
| --- | --- | --- | --- | --- |
| Map<String, Integer> broad.pda.seq.segmentation.AlignmentDataModel.getChromosomeLengths | ( |  | ) |  |

Implemented in broad.pda.seq.segmentation.GenericAlignmentDataModel.

Here is the caller graph for this function:

|  |  |  |  |  |  |
| --- | --- | --- | --- | --- | --- |
| CloseableIterator<Alignment> broad.pda.seq.segmentation.AlignmentDataModel.getChromosomeReadIterator | ( | String | *chromosome* | ) | throws IOException |

Parameters
:   |  |  |
    | --- | --- |
    | the | chromosome over which to iterate |

Returns
:   CloseableIterator over all reads mapped to the given Chromosome

Implemented in broad.pda.seq.segmentation.GenericAlignmentDataModel.

|  |  |  |  |  |
| --- | --- | --- | --- | --- |
| int broad.pda.seq.segmentation.AlignmentDataModel.getChunkEnd | ( |  | ) |  |

Implemented in broad.pda.seq.segmentation.GenericAlignmentDataModel.

|  |  |  |  |  |
| --- | --- | --- | --- | --- |
| int broad.pda.seq.segmentation.AlignmentDataModel.getChunkStart | ( |  | ) |  |

Implemented in broad.pda.seq.segmentation.GenericAlignmentDataModel.

|  |  |  |  |  |  |
| --- | --- | --- | --- | --- | --- |
| double broad.pda.seq.segmentation.AlignmentDataModel.getCounts | ( | String | *chr* | ) | throws IOException |

Counts the number of reads for the specified chromosome

Returns

Exceptions
:   |  |  |
    | --- | --- |
    | IOException |  |

Implemented in broad.pda.seq.segmentation.GenericAlignmentDataModel.

|  |  |  |  |
| --- | --- | --- | --- |
| double broad.pda.seq.segmentation.AlignmentDataModel.getCountsOfUniqueOverlappers | ( | Annotation | *target*, |
|  |  | Annotation | *exclude*, |
|  |  | IntervalTree< Alignment > | *tree*, |
|  |  | int | *EF* |
|  | ) |  |  |

Implemented in broad.pda.seq.segmentation.GenericAlignmentDataModel.

|  |  |  |  |
| --- | --- | --- | --- |
| double broad.pda.seq.segmentation.AlignmentDataModel.getCountsOfUniqueOverlappers | ( | Annotation | *target*, |
|  |  | Gene | *exclude*, |
|  |  | IntervalTree< Alignment > | *tree*, |
|  |  | int | *EF* |
|  | ) |  |  |

Implemented in broad.pda.seq.segmentation.GenericAlignmentDataModel.

|  |  |  |  |
| --- | --- | --- | --- |
| double broad.pda.seq.segmentation.AlignmentDataModel.getCountsOnBase | ( | String | *chr*, |
|  |  | int | *index* |
|  | ) |  | throws IOException |

Implemented in broad.pda.seq.segmentation.GenericAlignmentDataModel.

|  |  |  |  |
| --- | --- | --- | --- |
| double broad.pda.seq.segmentation.AlignmentDataModel.getCountsPerAlignment | ( | Annotation | *first*, |
|  |  | int | *EF* |
|  | ) |  | throws IOException |

Implemented in broad.pda.seq.segmentation.GenericAlignmentDataModel.

Here is the caller graph for this function:

|  |  |  |  |
| --- | --- | --- | --- |
| double broad.pda.seq.segmentation.AlignmentDataModel.getCountsPerAlignment | ( | Annotation | *align*, |
|  |  | IntervalTree< Alignment > | *tree*, |
|  |  | int | *EF* |
|  | ) |  |  |

Implemented in broad.pda.seq.segmentation.GenericAlignmentDataModel.

|  |  |  |  |
| --- | --- | --- | --- |
| double broad.pda.seq.segmentation.AlignmentDataModel.getCountsPerAlignment | ( | Gene | *gene*, |
|  |  | IntervalTree< Alignment > | *tree*, |
|  |  | int | *EF* |
|  | ) |  |  |

Implemented in broad.pda.seq.segmentation.GenericAlignmentDataModel.

|  |  |  |  |
| --- | --- | --- | --- |
| double broad.pda.seq.segmentation.AlignmentDataModel.getCountsPerAlignment | ( | Annotation[] | *Annotation*, |
|  |  | IntervalTree< Alignment > | *tree*, |
|  |  | int | *EF* |
|  | ) |  |  |

Implemented in broad.pda.seq.segmentation.GenericAlignmentDataModel.

|  |  |  |  |
| --- | --- | --- | --- |
| double broad.pda.seq.segmentation.AlignmentDataModel.getCountsPerAlignment | ( | Annotation[] | *Annotation*, |
|  |  | int | *EF* |
|  | ) |  | throws IOException |

Similar to

See also
:   getCountsPerAlignment(Annotation[] Annotation, IntervalTree<Alignment> tree, int EF) but delegates the management of the alignment interval tree to the implementing class rather than the caller

Parameters
:   |  |  |
    | --- | --- |
    | Annotation |  |
    | EF |  |

Returns

Exceptions
:   |  |  |
    | --- | --- |
    | IOException |  |

Implemented in broad.pda.seq.segmentation.GenericAlignmentDataModel.

|  |  |  |  |
| --- | --- | --- | --- |
| double broad.pda.seq.segmentation.AlignmentDataModel.getCountsPerAlignment | ( | Annotation | *align*, |
|  |  | Map< String, IntervalTree< Annotation >> | *goodExonTree*, |
|  |  | IntervalTree< Alignment > | *tree*, |
|  |  | int | *extensionFactor* |
|  | ) |  |  |

Implemented in broad.pda.seq.segmentation.GenericAlignmentDataModel.

|  |  |  |  |
| --- | --- | --- | --- |
| double broad.pda.seq.segmentation.AlignmentDataModel.getCountsPerAlignmentFullyContained | ( | Gene | *gene*, |
|  |  | IntervalTree< Alignment > | *tree*, |
|  |  | int | *EF* |
|  | ) |  |  |

Implemented in broad.pda.seq.segmentation.GenericAlignmentDataModel.

|  |  |  |  |
| --- | --- | --- | --- |
| double broad.pda.seq.segmentation.AlignmentDataModel.getCountsPerAnnotationtranded | ( | Gene | *gene*, |
|  |  | IntervalTree< Alignment > | *tree*, |
|  |  | int | *EF*, |
|  |  | String | *orientation* |
|  | ) |  |  |

Implemented in broad.pda.seq.segmentation.GenericAlignmentDataModel.

|  |  |  |  |
| --- | --- | --- | --- |
| int broad.pda.seq.segmentation.AlignmentDataModel.getCountsWithinExons | ( | Annotation | *align*, |
|  |  | Iterator< Alignment > | *iter*, |
|  |  | int | *EF* |
|  | ) |  |  |

Implemented in broad.pda.seq.segmentation.GenericAlignmentDataModel.

|  |  |  |  |
| --- | --- | --- | --- |
| int broad.pda.seq.segmentation.AlignmentDataModel.getCountsWithinExons | ( | Annotation | *align*, |
|  |  | IntervalTree< Alignment > | *tree*, |
|  |  | int | *EF* |
|  | ) |  |  |

Implemented in broad.pda.seq.segmentation.GenericAlignmentDataModel.

|  |  |  |  |  |  |
| --- | --- | --- | --- | --- | --- |
| Iterator<Annotation> broad.pda.seq.segmentation.AlignmentDataModel.getExonAnnotationOverlappingRegion | ( | Annotation | *align* | ) | throws IOException |

Implemented in broad.pda.seq.segmentation.GenericAlignmentDataModel.

|  |  |  |  |  |  |
| --- | --- | --- | --- | --- | --- |
| IntervalTree<Annotation> broad.pda.seq.segmentation.AlignmentDataModel.getFullIntervalTreeAsAnnotation | ( | String | *chr* | ) | throws IOException |

Implemented in broad.pda.seq.segmentation.GenericAlignmentDataModel.

|  |  |  |  |
| --- | --- | --- | --- |
| GeneCounts broad.pda.seq.segmentation.AlignmentDataModel.getGeneCounts | ( | Gene | *gene*, |
|  |  | int | *extensionFactor* |
|  | ) |  | throws IOException |

Implemented in broad.pda.seq.segmentation.GenericAlignmentDataModel.

|  |  |  |  |
| --- | --- | --- | --- |
| IntervalTree<Alignment> broad.pda.seq.segmentation.AlignmentDataModel.getIntervalTree | ( | String | *chr*, |
|  |  | int | *start*, |
|  |  | int | *end* |
|  | ) |  | throws IOException |

Implemented in broad.pda.seq.segmentation.GenericAlignmentDataModel.

|  |  |  |  |
| --- | --- | --- | --- |
| IntervalTree<Alignment> broad.pda.seq.segmentation.AlignmentDataModel.getIntervalTreeCached | ( | String | *chr*, |
|  |  | int | *start*, |
|  |  | int | *end* |
|  | ) |  | throws IOException |

Implemented in broad.pda.seq.segmentation.GenericAlignmentDataModel.

|  |  |  |  |
| --- | --- | --- | --- |
| IntervalTree<Alignment> broad.pda.seq.segmentation.AlignmentDataModel.getIntervalTreeTruncatedCached | ( | String | *chromosome*, |
|  |  | int | *start*, |
|  |  | int | *end* |
|  | ) |  | throws IOException |

Implemented in broad.pda.seq.segmentation.GenericAlignmentDataModel.

|  |  |  |  |  |
| --- | --- | --- | --- | --- |
| double broad.pda.seq.segmentation.AlignmentDataModel.getMinimumMappingQuality | ( |  | ) |  |

Implemented in broad.pda.seq.segmentation.GenericAlignmentDataModel.

|  |  |  |  |  |
| --- | --- | --- | --- | --- |
| String broad.pda.seq.segmentation.AlignmentDataModel.getModelFilePath | ( |  | ) |  |

Implemented in broad.pda.seq.segmentation.GenericAlignmentDataModel.

|  |  |  |  |  |  |
| --- | --- | --- | --- | --- | --- |
| double broad.pda.seq.segmentation.AlignmentDataModel.getNumberOfSplicedReads | ( | Annotation | *align* | ) | throws IOException |

Implemented in broad.pda.seq.segmentation.GenericAlignmentDataModel.

|  |  |  |  |  |  |
| --- | --- | --- | --- | --- | --- |
| Collection<Annotation> broad.pda.seq.segmentation.AlignmentDataModel.getOverlappingRegions | ( | String | *chr* | ) | throws IOException |

Implemented in broad.pda.seq.segmentation.GenericAlignmentDataModel.

|  |  |  |  |
| --- | --- | --- | --- |
| Gene broad.pda.seq.segmentation.AlignmentDataModel.getPeak | ( | Gene | *gene*, |
|  |  | Gene | *startCodon*, |
|  |  | IntervalTree< Alignment > | *tree*, |
|  |  | int | *EF* |
|  | ) |  |  |

Implemented in broad.pda.seq.segmentation.GenericAlignmentDataModel.

|  |  |  |  |  |
| --- | --- | --- | --- | --- |
| CloseableIterator<Alignment> broad.pda.seq.segmentation.AlignmentDataModel.getReadIterator | ( |  | ) |  |

Implemented in broad.pda.seq.segmentation.GenericAlignmentDataModel.

|  |  |  |  |  |  |
| --- | --- | --- | --- | --- | --- |
| CloseableIterator<Alignment> broad.pda.seq.segmentation.AlignmentDataModel.getReadIterator | ( | Annotation | *region* | ) | throws IOException |

Implemented in broad.pda.seq.segmentation.GenericAlignmentDataModel.

|  |  |  |  |
| --- | --- | --- | --- |
| double broad.pda.seq.segmentation.AlignmentDataModel.getScorePerAlignmentFromCache | ( | Gene | *window*, |
|  |  | IntervalTree< Alignment > | *chunkAlignmentTree*, |
|  |  | int | *extensionFactor* |
|  | ) |  |  |

Implemented in broad.pda.seq.segmentation.GenericAlignmentDataModel.

|  |  |  |  |  |  |
| --- | --- | --- | --- | --- | --- |
| Collection<? extends Annotation> broad.pda.seq.segmentation.AlignmentDataModel.getSplicedReadExons | ( | String | *chr* | ) | throws IOException |

Implemented in broad.pda.seq.segmentation.GenericAlignmentDataModel.

|  |  |  |  |
| --- | --- | --- | --- |
| Map<Annotation, Integer> broad.pda.seq.segmentation.AlignmentDataModel.getSplicedReads | ( | Annotation | *align*, |
|  |  | int | *minIntronSize*, |
|  |  | int | *maxIntronSize* |
|  | ) |  | throws IOException |

|  |  |  |  |
| --- | --- | --- | --- |
| Map<Annotation, Integer> broad.pda.seq.segmentation.AlignmentDataModel.getSplicedReads | ( | Annotation | *region*, |
|  |  | Collection< Predicate< Annotation >> | *filters* |
|  | ) |  | throws IOException |

Implemented in broad.pda.seq.segmentation.GenericAlignmentDataModel.

|  |  |  |  |  |  |
| --- | --- | --- | --- | --- | --- |
| Map<Annotation, Integer> broad.pda.seq.segmentation.AlignmentDataModel.getSplicedReads | ( | Annotation | *region* | ) | throws IOException |

Implemented in broad.pda.seq.segmentation.GenericAlignmentDataModel.

|  |  |  |  |
| --- | --- | --- | --- |
| Map<Annotation, Integer> broad.pda.seq.segmentation.AlignmentDataModel.getSplicedReads | ( | Annotation | *align*, |
|  |  | final int | *minIntronSize*, |
|  |  | final int | *maxIntronSize*, |
|  |  | int | *minNumIntrons*, |
|  |  | Sequence | *chrSeq* |
|  | ) |  | throws IOException |

Implemented in broad.pda.seq.segmentation.GenericAlignmentDataModel.

|  |  |  |  |
| --- | --- | --- | --- |
| Map<Annotation, Integer> broad.pda.seq.segmentation.AlignmentDataModel.getSplicedReads | ( | Annotation | *region*, |
|  |  | Collection< Predicate< Annotation >> | *filters*, |
|  |  | int | *minNumIntrons* |
|  | ) |  | throws IOException |

Implemented in broad.pda.seq.segmentation.GenericAlignmentDataModel.

|  |  |  |  |
| --- | --- | --- | --- |
| Map<Annotation, Integer> broad.pda.seq.segmentation.AlignmentDataModel.getSplicedReads | ( | Annotation | *region*, |
|  |  | Collection< Predicate< Annotation >> | *filters*, |
|  |  | int | *minNumIntrons*, |
|  |  | Sequence | *chrSeq* |
|  | ) |  | throws IOException |

Implemented in broad.pda.seq.segmentation.GenericAlignmentDataModel.

|  |  |  |  |
| --- | --- | --- | --- |
| Map<Annotation, Integer> broad.pda.seq.segmentation.AlignmentDataModel.getSplicedReads | ( | Annotation | *region*, |
|  |  | Predicate< Annotation > | *filter* |
|  | ) |  | throws IOException |

Implemented in broad.pda.seq.segmentation.GenericAlignmentDataModel.

|  |  |  |  |
| --- | --- | --- | --- |
| Map<Annotation, Integer> broad.pda.seq.segmentation.AlignmentDataModel.getSplicedReads | ( | int | *minIntronSize*, |
|  |  | int | *maxIntronSize* |
|  | ) |  | throws IOException |

Implemented in broad.pda.seq.segmentation.GenericAlignmentDataModel.

|  |  |  |  |  |
| --- | --- | --- | --- | --- |
| double broad.pda.seq.segmentation.AlignmentDataModel.getSpliceWeightFactor | ( |  | ) |  |

Implemented in broad.pda.seq.segmentation.GenericAlignmentDataModel.

|  |  |  |  |  |
| --- | --- | --- | --- | --- |
| double broad.pda.seq.segmentation.AlignmentDataModel.getTotalNumberOfStrandedReads | ( |  | ) |  |

Implemented in broad.pda.seq.segmentation.GenericAlignmentDataModel.

|  |  |  |  |  |  |
| --- | --- | --- | --- | --- | --- |
| boolean broad.pda.seq.segmentation.AlignmentDataModel.hasNextExon | ( | String | *chr* | ) | throws IOException |

Implemented in broad.pda.seq.segmentation.GenericAlignmentDataModel.

|  |  |  |  |  |
| --- | --- | --- | --- | --- |
| boolean broad.pda.seq.segmentation.AlignmentDataModel.isNegativeStranded | ( |  | ) |  |

Implemented in broad.pda.seq.segmentation.GenericAlignmentDataModel.

|  |  |  |  |  |
| --- | --- | --- | --- | --- |
| boolean broad.pda.seq.segmentation.AlignmentDataModel.isPositiveStranded | ( |  | ) |  |

Implemented in broad.pda.seq.segmentation.GenericAlignmentDataModel.

|  |  |  |  |  |
| --- | --- | --- | --- | --- |
| boolean broad.pda.seq.segmentation.AlignmentDataModel.isStranded | ( |  | ) |  |

Implemented in broad.pda.seq.segmentation.GenericAlignmentDataModel.

|  |  |  |  |
| --- | --- | --- | --- |
| boolean broad.pda.seq.segmentation.AlignmentDataModel.passes | ( | Gene | *gene*, |
|  |  | IntervalTree< Alignment > | *tree*, |
|  |  | int | *EF*, |
|  |  | double | *cutoff* |
|  | ) |  |  |

Implemented in broad.pda.seq.segmentation.GenericAlignmentDataModel.

|  |  |  |  |
| --- | --- | --- | --- |
| long broad.pda.seq.segmentation.AlignmentDataModel.passesCutoff | ( | Annotation | *align*, |
|  |  | IntervalTree< Alignment > | *tree*, |
|  |  | int | *EF*, |
|  |  | double | *threshold* |
|  | ) |  |  |

Implemented in broad.pda.seq.segmentation.GenericAlignmentDataModel.

|  |  |  |  |  |
| --- | --- | --- | --- | --- |
| void broad.pda.seq.segmentation.AlignmentDataModel.resetTreeCache | ( |  | ) |  |

Implemented in broad.pda.seq.segmentation.GenericAlignmentDataModel.

|  |  |  |  |  |  |
| --- | --- | --- | --- | --- | --- |
| void broad.pda.seq.segmentation.AlignmentDataModel.resetTreeCache | ( | String | *chr* | ) |  |

Implemented in broad.pda.seq.segmentation.GenericAlignmentDataModel.

|  |  |  |  |  |
| --- | --- | --- | --- | --- |
| void broad.pda.seq.segmentation.AlignmentDataModel.restartIterator | ( |  | ) |  |

Implemented in broad.pda.seq.segmentation.GenericAlignmentDataModel.

|  |  |  |  |  |  |
| --- | --- | --- | --- | --- | --- |
| void broad.pda.seq.segmentation.AlignmentDataModel.setChunkSize | ( | int | *chunkSize* | ) |  |

Implemented in broad.pda.seq.segmentation.GenericAlignmentDataModel.

|  |  |  |  |  |  |
| --- | --- | --- | --- | --- | --- |
| void broad.pda.seq.segmentation.AlignmentDataModel.setExtensionFactor | ( | int | *factor* | ) |  |

Implemented in broad.pda.seq.segmentation.GenericAlignmentDataModel.

|  |  |  |  |  |
| --- | --- | --- | --- | --- |
| void broad.pda.seq.segmentation.AlignmentDataModel.setFirstRead | ( |  | ) |  |

Implemented in broad.pda.seq.segmentation.GenericAlignmentDataModel.

|  |  |  |  |  |  |
| --- | --- | --- | --- | --- | --- |
| void broad.pda.seq.segmentation.AlignmentDataModel.setMinimumMappingQuality | ( | double | *minimumMapQuality* | ) |  |

Implemented in broad.pda.seq.segmentation.GenericAlignmentDataModel.

|  |  |  |  |  |
| --- | --- | --- | --- | --- |
| void broad.pda.seq.segmentation.AlignmentDataModel.setNegativeStranded | ( |  | ) |  |

Implemented in broad.pda.seq.segmentation.GenericAlignmentDataModel.

|  |  |  |  |  |  |
| --- | --- | --- | --- | --- | --- |
| void broad.pda.seq.segmentation.AlignmentDataModel.setNormalizationStrategy | ( | ReadCountNormalizer | *normalizer* | ) |  |

Implemented in broad.pda.seq.segmentation.GenericAlignmentDataModel.

|  |  |  |  |  |
| --- | --- | --- | --- | --- |
| void broad.pda.seq.segmentation.AlignmentDataModel.setPositiveStranded | ( |  | ) |  |

Implemented in broad.pda.seq.segmentation.GenericAlignmentDataModel.

|  |  |  |  |  |
| --- | --- | --- | --- | --- |
| void broad.pda.seq.segmentation.AlignmentDataModel.setSecondRead | ( |  | ) |  |

Implemented in broad.pda.seq.segmentation.GenericAlignmentDataModel.

|  |  |  |  |  |
| --- | --- | --- | --- | --- |
| void broad.pda.seq.segmentation.AlignmentDataModel.unsetStranded | ( |  | ) |  |

Implemented in broad.pda.seq.segmentation.GenericAlignmentDataModel.

|  |  |  |  |
| --- | --- | --- | --- |
| Gene broad.pda.seq.segmentation.AlignmentDataModel.updateGeneByFirstCounts | ( | Gene | *gene*, |
|  |  | IntervalTree< Alignment > | *tree*, |
|  |  | int | *EF* |
|  | ) |  |  |

Implemented in broad.pda.seq.segmentation.GenericAlignmentDataModel.

---

The documentation for this interface was generated from the following file:

- java/broad/pda/seq/segmentation/AlignmentDataModel.java


---

Generated on Fri Aug 15 2014 15:11:04 for ESAT by  

 1.8.7
